# Could kDNA-PCR in Peripheral Blood Replace the Examination of Bone Marrow for the Diagnosis of Visceral Leishmaniasis?

**DOI:** 10.1155/2016/1084353

**Published:** 2016-08-14

**Authors:** Natalia Souza de Godoy, Marcos Luiz Alves Andrino, Regina Maia de Souza, Erika Gakiya, Valdir Sabbaga Amato, José Ângelo Lauletta Lindoso, Lucia Maria Almeida Braz

**Affiliations:** ^1^Laboratório de Investigação Médica, Parasitologia LIM 46 do Hospital das Clínicas da Faculdade de Medicina da Universidade de São Paulo (HCFMUSP), Avenida Dr. Enéas Carvalho de Aguiar 470/500, 05403-000 São Paulo, SP, Brazil; ^2^Departamento de Moléstias Infecciosas e Parasitárias, HCFMUSP, Avenida Dr. Enéas Carvalho de Aguiar 255, 05403-000 São Paulo, SP, Brazil; ^3^Laboratório de Soroepidemiologia e Imunobiologia, IMTSP-USP, Avenida Dr. Enéas Carvalho de Aguiar 470/500, 05403-000 São Paulo, SP, Brazil; ^4^Hospital Emilio Ribas, Avenida Dr. Arnaldo 165, 01246-900 São Paulo, SP, Brazil; ^5^Laboratório de Parasitologia, Instituto de Medicina Tropical de São Paulo, Universidade de São Paulo (IMTSP-USP), Avenida Dr. Enéas Carvalho de Aguiar 470/500, 05403-000 São Paulo, SP, Brazil

## Abstract

The aim of this study was to evaluate whether the molecular (kDNA-PCR) and parasitological diagnosis in peripheral blood (PB) could replace the invasive and painful bone marrow collection (BM) in the diagnosis of visceral leishmaniasis (VL). PB from suspected VL patients was evaluated by parasitological and molecular techniques using as the gold standard (GS) a combination of clinical, epidemiological, and immunochromatographic test (PB-rK39) results and parasitological examination of BM. Based on the GS, 38 samples from 32 patients were grouped: Group 1, 20 samples of VL cases, and Group 2, 18 samples of non-VL cases. In order to evaluate the parasitological and molecular techniques in PB, the samples were examined. From Group 1, PB kDNA-PCR was positive in 20 samples and in 19 of 20 in BM kDNA-PCR examination. However, the parasitological examination of buffy coat was insensitive, being able to detect only 4 cases from Group 1. All samples from Group 2 were negative. We concluded that, for the diagnosis of visceral leishmaniasis, the parasitological examination of peripheral blood was not useful; however, molecular diagnosis by kDNA-PCR, performed in peripheral blood, could be useful to replace the parasitological examination of bone marrow.

## 1. Introduction

Visceral leishmaniasis (VL) has been reported in 88 countries, and 90% of the world's burden is localized in India, Brazil, and Sudan [1]. In the Americas, VL is known as American Visceral Leishmaniasis (AVL) and its etiologic agent is* Leishmania* (*Leishmania*)* infantum* (*L.* (*L.*)* chagasi*, syn.) [2]. VL is characterized by its chronicity and systemic dissemination capacity [3] and it can be fatal if treatment is not administered. To be correctly diagnosed, VL requires the use of highly sensitive and specific laboratory methods [4, 5]. In the context of VL, the techniques that are considered gold standards (GS) are the direct microscopic examination of bone marrow (BM) or spleen aspirate samples, with observation of amastigotes on smears (BM-S), and the isolation of promastigotes in culture (BM-C) [6, 7]. More recently, the rK39 immunochromatographic test has been included [8]. Although splenic aspirate smears show the highest sensitivity, followed by BM aspirate smears, both exams require invasive and more risky procedures [9–11]. Given the fact that a considerable number of VL patients are children and immunocompromised patients, it would be desirable to adopt less risky and painful procedures provided that similar sensitivity and specificity rates are obtained. Therefore, the aim of this study was to evaluate whether parasitological and molecular techniques performed in the BC (buffy coat) or WB (whole blood), from a small volume of peripheral blood (PB), could replace the parasitological examination of the bone marrow (BM), whose collection is invasive and painful, for the diagnosis of visceral leishmaniasis (VL).

Peripheral blood (PB) is a biological material that can be easily collected and used in highly sensitive tests to investigate VL such as* Leishmania* DNA amplification by PCR performed in whole blood or buffy coat samples [4] and serological detection of anti-*Leishmania* antibodies [6].

Using samples of whole blood (WB) and buffy coat (BC) from patients with and without VL, parasitological and molecular techniques were evaluated. These samples were used to prepare smears (PB-S) and inoculated in NNN medium supplemented with BHI (PB-C). In addition, a kDNA-PCR was performed in WB and BC samples (PB kDNA-PCR) as well as in BM samples (BM kDNA-PCR). The performances of these techniques were compared to the GS laboratorial techniques (PB-rK39; BM-S and BM-C) results.

## 2. Methods

### 2.1. Patients

This research was approved by the Institutional Research Ethics Committee (protocol number 0006/11). After signing the informed consent, participants suspected to have VL coming from different regions of Brazil were attended at the University Hospital (HC-FMUSP) of São Paulo. From 77 samples examined, we selected 38 samples from 32 patients, whose samples from BM and PB were matched.

### 2.2. Laboratorial Gold Standards (GS) and Definition of VL Cases

The laboratorial GS were the parasitological techniques (direct microscopy examination and culture) of BM samples, aside from the immunochromatographic diagnostic test (rK39) in whole blood PB samples (PB-rK39). To be considered a true VL case the participant had to present clinical test, epidemiological test, and at least one positive test among the laboratorial GS.

Thirty-eight samples from 32 patients met the inclusion criteria of the study and were grouped as follows:

 Group 1: 20 samples from 18 true VL cases according to the clinical and epidemiological data associated with a positive result in at least one GS laboratorial techniques.
 Group 2: 18 samples from 16 non-VL cases presented symptoms initially compatible with VL and had negative results in the GS laboratorial techniques.

### 2.3. Collection and Processing of Biological Samples

BM aspirates and PB samples (3.5 mL) were collected in EDTA tubes and submitted to the following diagnostic procedures.

#### 2.3.1. Parasitological Techniques (Gold Standard When Tested in Bone Marrow)

*Microscopy Examination of the Stained Smear from Bone Marrow (BM-S) and from Peripheral Blood Samples PB (PB-S)*. Five microliters of BM and PB samples (WB and BC at 1,506 ×g and 20,000 ×g, resp.) was used to prepare eight slides smears. From each procedure (BM, WB PB, and BCPB1 at 1,506 ×g and BCPB2 at 20,000 ×g) 2 slides were prepared, which were subsequently stained by Panótico method (Newprov Instant Prov, Paraná, Brazil), a kind of Leishman or Romanowsky dye. They were analyzed by microscopy (1000x magnification), searching for amastigotes, and 200 fields were examined in each smear [12]. 

*Culture of BM (BM-C) and PB (PB-C) in NNN Medium Supplemented with BHI.* Forty microliters of BM or the same volume of BC (1,506 ×g and 20,000 ×g) from PB samples was inoculated into 2 tubes, containing NNN medium (DIFCO, USA), to which 2 mL of BHI medium was added (DIFCO, USA). The tubes were incubated in a BOD incubator at 25°C and the samples were weekly analyzed for a total of 30 days by means of optical microscopy searching for promastigotes [13].

#### 2.3.2. Molecular Diagnostic Techniques of Peripheral Blood (PB) and Bone Marrow (BM) Polymerase Chain Reaction: PB kDNA-PCR and BM kDNA-PCR

WB and BC obtained by centrifugation (1,506 ×g and 20,000 ×g) of PB samples as well as BM samples were submitted to DNA extraction with the aid of the Genomic DNA Extraction Kit (Real Biotech Corporation, Taiwan, China) starting with an initial volume of 300 *μ*L. DNA samples were identified and stored at −20°C. The kDNA primers were designed within a conserved region of* Leishmania* sp. kDNA minicircles. The forward primer 20 (5′-GGGKAGGGGCGTTCTSCGA A-3′) and reverse primer 22 (5′-SSSWCTATWTTACACCAACCCC-3′) yielded a 120-base-pair amplification product [14].

Amplifications were performed in a total volume of 20 *μ*L containing 100 ng of template DNA, 50 mM of KCl, 10 mM of Tris-HCl (pH 8.0), 0.2 mM of dNTPs (Fermentas, Thermo Fisher, Ontario, Canada), 1.0 mM of MgCl_2_, 0.4 *μ*M of each primer, and 1 unit of Taq DNA Polymerase (Fermentas, Thermo Fisher, Ontario, Canada). In each experiment, two negative controls containing sterile water instead of template DNA were also tested. The positive control was* Leishmania infantum* DNA extracted from cultures. Each reaction was performed with an initial denaturation step of 94°C for 5 minutes, followed by 35 cycles of 94°C for 1 minute, 58°C for 1 minute, and 72°C for 30 seconds, ending with a final extension step of 72°C for 5 minutes. Reactions were performed in a minicycler thermocycler (MJ Research Corp/MJ Research, Quebec, Canada) and PCR products were visualized in 2% ethidium bromide-stained agarose gels by means of a UV transilluminator (Alpha Innotech Corp/Alpha Innotech Multimage, California, USA).

To minimize the risk of contamination, reagents preparation and PCR master mix, DNA extraction, and electrophoresis were performed in three separate areas. To confirm that amplification inhibitors were not present, a fragment of the human beta-globin gene was tested in all the samples [15].

#### 2.3.3. Serological Technique

Whole PB samples were tested by the PB-rK39 immunochromatographic kit that uses a recombinant peptide containing 39 amino acid repeats from the kinesin-like gene found in* L. chagasi*. This test is widely used for VL diagnosis in field studies [11].

### 2.4. Statistical Analysis

To determine the agreement of tests, the kappa test was used at a 95% confidence interval and *p* values ≤0.05 were considered significant. The data were analyzed with the statistical software STATA version 12.0 (Stata Corp LP, College Station, Texas, USA).

## 3. Results 

Regarding the GS techniques (Table 1), performed in Group 1 (20 true VL samples), we obtained the following results: 16 samples were positive by BM-S, including 8 also by BM-C; 15 were positive by PB-rK39 (four of which were negative by parasitological examination of BM), though there were five samples, from two HIV positive patients, that yielded negative results by PB-rK39 and were positive by parasitological examination of BM. Group 2 was composed of 18 samples from 16 non-VL cases that were negative by all the gold standard techniques (BM-S, BM-C, and PB-rK39). The clinical follow-up of these 16 non-VL cases revealed, in most of them, cutaneous leishmaniasis (1), lupus (2), prostatitis (1), hepatitis (1), urinary tract infection (1), Sjogren syndrome (1), lymphoma (2), gastric ulcers (2), sarcoidosis (1), pellagra (1), pharyngitis (1), hypothyroidism (1), and anemia (1). All DNA samples were amplified by the beta-globin gene demonstrating that there were no amplification inhibitors.

According to Table 1, PB kDNA-PCR, performed in whole blood and buffy coat, was positive in 100% of Group 1 samples (20/20). PB-S was positive in 20% of the samples (4/20), and PB-C was negative in all samples. The four PB-S positive samples were obtained from BCPB1 (one buffy coat sample centrifuged at 1,506 ×g) and BCPB2 (two buffy coat samples centrifuged at 20,000 ×g) and one in BCPB1 and BCPB2 (buffy coat sample tested after centrifugation at 1,506 ×g and 20,000 ×g). It is noteworthy that none of these four samples was parasitologically positive when the analysis was performed directly in the corresponding whole blood samples.

Analyzing the PB kDNA-PCR and BM kDNA-PCR they presented a specificity of 100% and a sensitivity of 100% and 95%, respectively (Table 2).

A good concordance, with a kappa index of 0,79 and 0,74, was obtained (*p* < 0.001), when PB kDNA-PCR was compared with the gold standard techniques: the BM-S and the rK39, respectively (Table 3). Comparison between PB kDNA-PCR and BM kDNA-PCR results showed an almost perfect correlation (0.94, *p* < 0.001) (Table 3).

## 4. Discussion 

In this study PB samples were tested as a substitute of invasive and painful procedures to obtain BM samples for the diagnosis of VL. Abeijon and Campos-Neto [16] investigated a potential noninvasive urine-based antigen detection assay to diagnose active VL. One of the advantages of PB samples to investigate VL is that the same biological material can be concomitantly analyzed by parasitological, serological, and molecular techniques unlike urine samples. The main symptoms of VL patients in our study were hepatosplenomegaly, febrile, and pancytopenia as presented for all the patients in Group 1 (true VL cases, Table 1). They were all Brazilians and came from VL endemic regions [2, 3].

Regarding the parasitological investigation in peripheral blood, buffy coat PB-S detected 20% of positive samples, while none of the samples were positive when whole blood samples were examined, as expected, because parasites are concentrated in buffy coat facilitating the visualization of amastigotes within leucocytes [4]. Nevertheless, 20% detection is very low when compared to BM-S, one of the gold standards (80% of positivity). Similarly, PB-C did not find any positive sample while BM-C detected 40%. Sixteen out of 20 samples were negative by PB-S, and this low sensitivity can be explained by the fact that there are more parasites in the bone marrow and spleen samples [6, 7], justifying why they are the gold standard of VL laboratorial investigation. In the present study, cultures were not a sensitive method compared to other techniques, even in the case of BM, as this exam was able to confirm only 40% of truly positive samples. Aside from being very prone to contamination, cultures are time consuming requiring four weeks to release a final result, what has already been acknowledged by other authors [17] and was corroborated in this research.

According to the ideal molecular target for detecting* Leishmania*, kDNA was chosen due to the large presence of minicircles in the cells of the parasite, about 10^4^ copies per cell [17]. Moreover, the proven viability of parasites in PB, which is a biological material, obtained more easily than the BM leads us to search for kDNA in the PB. Nevertheless, differently of parasitological investigation in PB, molecular investigation (kDNA) in PB did not present differences between buffy coat (BCPB: 1506 ×g and BCPB: 20000 ×g) and whole blood. All samples 20/20 were positive. According to Srivastava et al. [4] PCR analysis of the whole blood or its buffy coat preparation may prove a useful screening test. A sensitivity of 100% was obtained by PB kDNA-PCR when compared to the gold standard techniques (BM-S, BM-C, and PB-rK39). By comparing molecular and parasitological techniques, Ozerdem et al. [18] obtained better results with kDNA-PCR (29/50 or 58%) in comparison with microscopic examination (10/50 or 20%) of Giemsa-stained smears from blood samples of suspected VL patients. In our study, PB kDNA-PCR showed a good concordance with the rapid immunochromatographic test (PB-rK39) (0.74, *p* < 0.001), which is a rapid and highly sensitive technique, so that it has been used as a reference test. Disch et al. [9] and Andresen et al. [19] obtained sensitivities of 91% (48/53) and 92.5% (37/40) when kDNA-PCR was used to test whole blood from PB samples of patients with VL, confirmed by clinical and microscopic examination of BM or lymph node samples. Fraga et al. [20] evaluated the effectiveness of a kDNA-PCR in PB and found a very good sensitivity (43/45; 95.6%), which was higher than that found in BM samples: kDNA (41/45; 91.1%); microscopic examination of smear (36/45; 80%); and culture (12/45; 26.7%). Antinori et al. [21] used PCR and obtained a sensitivity of 98.5% (64/65) and 95.7% (45/47) in PB and BM, respectively, once again confirming the better sensitivity of PB-PCR (whole blood) in comparison with BM-PCR. In contrast, Cruz et al. [10] found a higher positivity of Ln-PCR in BM (24/24) than in whole blood of PB (Ln-PCR) (19/24).

By means of immunochromatography test, due to its sensitivity and rapidity, the PB-rK39 can screen suspected cases of VL, especially in immunocompetent patients. Although it is not our main objective, it is necessary to reinforce that the presence of VL antibodies, associated with clinical and epidemiological data, can assist in prompt medical decisions, but it cannot differentiate past from active infection. In order to diagnose an active VL infection in PB samples, from immunocompetent or immunodeficient patients, kDNA-PCR is the most appropriate. Unlike the BM, it also presents the advantage of being an easy to take biological sample. Nonetheless, parasitological examination of the peripheral blood (PB-S and PB-C) cannot substitute the parasitological examination of bone marrow (BM-S and BM-C).

In conclusion, kDNA-PCR performed in small volumes of PB, in either whole blood or buffy coat, showed a good agreement with VL gold standard tests. Therefore peripheral blood could be useful to replace the invasive and painful procedures to obtain bone marrow samples for the diagnosis of visceral leishmaniasis.

## Figures and Tables

**Table 1:** Results of clinical, epidemiological, and laboratorial diagnosis of visceral leishmaniasis in Group 1 (true VL cases) (1).

Number of samples (*N*), age (*A*), and sample entrance (SE)	Comorbidities (CM)	Clinical and laboratorial manifestations	Epidemiological data (place of origin)	Laboratorial investigation for VL (gold standard)			Laboratorial investigation for VL (tests)			
				Parasitological techniques in bone marrow (BM)		Serological technique (rK39) in peripheral blood (PB)	Parasitological techniques in peripheral blood (PB)		Molecular technique (kDNA) in bone marrow (BM) and peripheral blood (PB)	
				BM-S	BM-C	PB-rK39	PB-S	PB-C	BM-kDNA	PB-kDNA

*N*: 1.1 *A*: 19 SE: 07/30/2009	Systemic lupus erythematosus	Hepatosplenomegaly and pancytopenia	Bahia Sister and dog with VL	Neg	Neg	Pos	Neg	Neg	Pos	Pos
*N*: 1.2 *A*: 20 SE: 05/25/2011	Systemic lupus erythematosus	VL with death Hepatosplenomegaly and pancytopenia	Bahia	Pos	Neg	Pos	Neg	Neg	Pos	Pos
*N*: 2.1 *A*: 31 SE: 08/14/2009	HIV	Hepatosplenomegaly and fever	Mato Grosso	Pos	Neg	Neg	Neg	Neg	Pos	Pos
*N*: 2.2 *A*: 33 SE: 02/22/2011	HIV	Hepatosplenomegaly and fever	Mato Grosso	Pos	Neg	Neg	Neg	Neg	Pos	Pos
*N*: 2.3 *A*: 33 SE: 03/11/2011	HIV	Hepatosplenomegaly and fever	Mato Grosso	Pos	Neg	Neg	Neg	Neg	Pos	Pos
*N*: 2.4 *A*: 33 SE: 03/30/2011	HIV	Hepatosplenomegaly and fever	Mato Grosso	Pos	Neg	Neg	Neg	Neg	Pos	Pos
*N*: 3 *A*: 26 SE: 09/18/2009	HIV	VL severe with death Hepatosplenomegaly and fever	São Paulo	Pos	Neg	Neg	Neg	Neg	Neg	Pos

*N*: 4 *A*: 69 SE: 12/07/2009		Hepatosplenomegaly	Ceará	Neg	Neg	Pos	Neg	Neg	Pos	Pos
*N*: 5 *A*: 30 SE: 02/01/2010	No	Hepatosplenomegaly, fever, and weight of loss	Bahia	Neg	Neg	Pos	Neg	Neg	Pos	Pos
*N*: 6 *A*: 74 SE: 04/16/2010	No	VL with death Pancytopenia and fever	Minas Gerais	Pos	Pos	Pos	Neg	Neg	Pos	Pos
*N*: 7 *A*: 40 SE: 11/16/2010	No	Splenomegaly, plateletopenia, anaemia, and fever	Pernambuco	Pos	Pos	Pos	Neg	Neg	Pos	Pos
*N*: 8 *A*: 29 SE: 05/11/2011	Schistosomiasis	Hepatosplenomegaly and fever	Bahia	Pos	Pos	Pos	Pos	Neg	Pos	Pos
*N*: 9 *A*: 65 SE: 06/28/2011	Hemophagocytic syndrome	Hepatosplenomegaly, fever, and pancytopenia	Bahia	Pos	Pos	Pos	Pos	Neg	Pos	Pos
*N*: 10 *A*: 53 SE: 08/23/2011	Schistosomiasis hanseniasis	Pancytopenia	Alagoas	Pos	Pos	Pos	Pos	Neg	Pos	Pos
*N*: 11 *A*: 33 SE: 08/23/2011	No	Hepatosplenomegaly and fever	Bahia	Pos	Pos	Pos	Neg	Neg	Pos	Pos
*N*: 12 *A*: 4 SE: 08/16/2011	No	Hepatosplenomegaly, fever pancytopenia, anaemia, weight loss, and lymphadenomegaly	Mato Grosso	Neg	Neg	Pos	Neg	Neg	Pos	Pos

*N*: 13 *A*: 2 SE: 08/25/2011	No	Hepatosplenomegaly, fever, and pancytopenia	Piauí	Pos	Pos	Pos	Pos	Neg	Pos	Pos
*N*: 14 *A*: 48 SE: 08/29/2011	No	Hepatosplenomegaly, fever, and pancytopenia	Bahia	Pos	Neg	Pos	Neg	Neg	Pos	Pos
*N*: 15 *A*: 45 SE: 11/11/2011	No	Hepatosplenomegaly, fever, and weight loss	Bahia	Pos	Pos	Pos	Neg	Neg	Pos	Pos
*N*: 16 *A*: 20 SE: 03/27/2011	No	Hepatosplenomegaly and fever	Bahia	Pos	Neg	Pos	Neg	Neg	Pos	Pos

BM-S: smear with sample from bone marrow; BM-C: culture with sample from bone marrow; PB-S: smear with sample from peripheral blood; PB-C: culture with sample from peripheral blood; PB-rK39: rK39 with sample from peripheral blood; BM-kDNA: kDNA PCR in sample from bone marrow; PB-kDNA: kDNA PCR in sample from peripheral blood; pos: positive; neg: negative.

**Table 2:** Sensitivity, specificity, predictive positive value, predictive negative value, probability of false positive, probability of false negative of PB kDNA-PCR and BM kDNA-PCR.

Test	Sensitivity (%)	Specificity (%)	PPV (%)	PNV (%)	PFP (%)	PFN (%)	Efficiency (%) (CI 95%)
PB kDNA-PCR	100	100	100	100	0	0	100 (90.7–100)
BM kDNA-PCR	95.0	100	100	95.2	0	4.8	97.3 (86.2–99.9)

BM kDNA-PCR: kDNA PCR in sample from bone marrow; PB kDNA-PCR: kDNA PCR in sample from peripheral blood; PPV: predictive positive value; PNV: predictive negative value; PFP: probability of false positive; PFN: probability of false negative; CI 95%: 95% confidence intervals.

**Table 3:** Comparative analysis between the results of PB kDNA-PCR and results of BM kDNA-PCR, BM-S, BM-C, and PB-rk39.

Test	PB kDNA-PCR			Kappa (CI 95%)	*p* value	Efficiency (%) (CI 95%)
	Positive	Negative	Total			
BM kDNA-PCR				0.947	**<0.001**	97.4 (86.2–99.9)
*Positive*	19	0	19	(0.846–1.000)		
*Negative*	1	18	19	Almost perfect correlation		
*Total*	20	18	38			

BM-S				0.791	**<0.001**	89.5 (75.2–97.1)
*Positive*	16	0	16	(0.602–0.980)		
*Negative*	4	18	22	Good concordance		
*Total*	20	18	38			

BM-C				0.387	**0.001**	68.4 (51.3–82.5)
*Positive*	8	0	8	(0.160–0.614)		
*Negative*	12	18	30	Low concordance		
*Total*	20	18	38			

PB-rK39				0.740	**<0.001**	86.4 (71.9–95.6)
*Positive*	15	0	15	(0.534–0.945)		
*Negative*	5	18	23	Good concordance		
*Total*	20	18	38			

BM kDNA-PCR: kDNA PCR in sample from bone marrow; PB kDNA-PCR: kDNA PCR in sample from peripheral blood; PB-rk39: rK39 with sample from peripheral blood; CI 95%: 95% confidence intervals.
